# 

**Published:** 1842-04-02

**Authors:** 


UNIVERSITY OF PENNSYLVANIA.—At a Public Commencement held the 26th of March, 1842, the following gentlemen received the Degree of Doctor of Medicine. An address was then delivered by Dr. Gibson, Professor of Surgery.

*North Carolina.*—John Q. Anderson, Cynanche Trachealis; William H. Banks, Puerperal Peritonitis; Oscar F. Baxter, Parturition; Jesse G. Bryan, Dysentery; William W. Clements, Intermittent Fever; Edward A. Crudup, Spontaneous Hæmorrhages; Shelby Currie, Dyspepsia; William W. Davis, Acute Gastritis; William J. Hawkins, Indigestion; Augustus H. Macnair, Menstruation; Robert J. Steele, Phthisis; Albert C. Wharton, The Revulsive Action of Medicine; Henry Yancey, Emetics.

*Alabama.*—Thomas J. Anderson, on Hygiene; James A. Dozier, Hydrocele; Joseph M. Heard, Depletion; Jesse E. Trippe, Acute Gastritis.

*Virginia.*—Henry Ashton, Intermitting Fever; Monro Banister, Puerperal Fever; William H. Batte, Dysentery; Walter A. Brown, Cynanche Trachealis; Patrick H. Christian, Disease of Kidney; Daniel Conrad, Acute Gastritis; William T. Cornick, Scarlatina; Robert W. Dailey, Amenorrhœa; John J. Gravatt, Scarlatina; Asa W. Graves, Hæmatemesis; James W. Green, Fractures; Solon P. C. Henkel, Aneurism; Josiah N. Jones, Intermittent Fever; Richard Kennon, Means of easing the pains of Parturition; Josiah Manry, Acute Gastritis; David E. Meade, Emotional Tears; John H. Mettert Leucorrhœa; Lewis A. Miller, Scarlatina; Hollowell Old, Peritonitis Puerperalis; Mann A. Page, Scarlatina; William P. Palmer, Irritable Uterus; Edward H. Pritchett, Metritis; Thomas C. Revely, Neuralgia; Moore Robinson, Pathology and Symptoms of Pneumonia; William W. Roper, Amenorrhœa; William Strother, Dyspepsia; John G. Tannor, Acute Gastritis; Francis Otway Tompkins, Menstruation; Robert B. Tunstall, Delirium Tremens; James A. Waddell, Iodine; Richard P. Walton, Arsenic; Francis West, Iron; John F. White, Prolapsus Uteri; John J. Wright, Fractures; John A. Hunter, Theory of Menstruation and Amenorrhœa; Wm. W. Gwathmey, Bilious Remittent Fever.

*Delaware.*—James V. Z. Blaney, The Invest. of the Veg. Mat. Med.; Saulsbury Gove, Rheumatism.

*New Jersey.*—Philip F. Brakeley, Anascara; Andrews E. Budd, Prolapsus Uteri; Lewis C. Cook, Reproduction; Theodore Johnes, Hydrophobia.

*Georgia.*—William A. Brinson, Congestive Fever of the South; Jeremiah Hilsman, Gonorrhœa.

*Ohio.*—John L. Burtt, Dietetics.

*Tennessee.*—Edward R. Cage, Cholera Infantum; John Dickinson, Delirium Tremens; Samuel M. Edgar, Gunshot Wounds; Edward B. Littlefield, Deformity from Anchylosis; James P. M'Farland, Fungus Hæmatodes; Walter S. M'Nairy, Intermittent Fever; John S. Peete, De Febre Biliosa Remittente; James W. Phillips, Idiosyncrasy; Benjamin F. Taliaferro, Hydrocephalus.

*Maryland.*—Samuel Chamberlaine, Urethral Stricture; George Dennis, Typhus Fever; Edwin W. Henry, Cholera Infantum; Henry H. Mitchell, Cholera Infantum; George W. Purnell, Icterus; William G. Rider, Tenotomy; Samuel Tyler, De Puerperali Peritonite; Thomas W. Woodland, Dyspepsia.

*Nova Scotia.*—Robert Dickey, Acute Peritonitis.

*St. Croix.*—John F. Egan, Blood.

*Philadelphia.*—Richard M. Greenbank, Autumnal Fever of Q. Ann Co.; Samuel L. Hollingsworth, Iritis; Elisha Kent Kane, Kyestine; Edward H. Ward, Amaurosis.

*Rhode Island.*—George A. Hammett, Akenesic Power.

*Missouri.*—James Hogan, Prolapsus Uteri.

*Kentucky.*—James M. Hood, Circulation of the Blood; James M. Reid, Scarlatina; William Todd, Inflammation.

*Pennsylvania.*—J. Temple Hotchkiss, Rubeola; Mahlon P. Hutchinson, Prolapsus Uteri; R. Parker Little, Tetanus; John Dickinson Logan, Arthritis; Franklin B. Martin, Pleuritis; Matthias J. Pennypacker, Respiration and Animal Heat; Griffith J. Scholl, Phlegmasia Dolens; Edwin Shoemaker, Acute Gastritis.

*Mississippi.*—Alexander C. L. Magruder, Congestive Fever of Mississippi; Thomas H. Young Aneurism.

*New York.*—John K. Mason, Scrofula; Harvey F. Montgomery, Dislocation of the Os Humeri.

*Louisiana.*—John Postlethwaite, Fever; Thomas Towles, Scarlatina.

*Massachusetts.*—George Shove, Therapeutics, John B. Walker, Hydrocele.

*Missouri.*—Richard Henry Stevens, Milk-sickness of the West.

*Florida.*—Thomas B. Taylor, Spermorrhœa.

At the Commencement in the Arts, held in July, 1841, the following gentlemen received the Degree of Doctor of Medicine.

*Georgia.*—Lorenzo N. Burge, Conception.

*Pennsylvania.*—John T. Clarke, Gonorrhœa; Maurice P. Linton, The Seven Eras of Women; John Miller, Intermittent Fever.

*Virginia.*—William M. Thompson, Acute Gastritis,

Total, 114. W. E. HORNER, Dean of Medical Faculty.

